# DlgR2 knockdown boosts dendritic cell activity and inhibits hepatocellular carcinoma tumor *in-situ* growth

**DOI:** 10.18632/oncotarget.18990

**Published:** 2017-07-05

**Authors:** Zhen Lu, Yun-Hong Xia, Min Zhao, Bing Zhang, Wen-Ting Dai, Lu Ding, Li-Xia Hu, Jin-Ling Bi, Guo-Lin Jiang

**Affiliations:** ^1^ Department of General Surgery, The Fourth Affiliated Hospital, Anhui Medical University, Hefei, China; ^2^ Department of Oncology, The Fourth Affiliated Hospital, Anhui Medical University, Hefei, China; ^3^ Hefei Hospital, Anhui Medical University, Hefei, China; ^4^ Key Laboratory of Anhui Medical University, Hefei, China

**Keywords:** hepatocellular carcinoma (HCC), dendritic cells, DIgR2, tumor immunity, oncotarget

## Abstract

Tumor-specific hepatic stellate cells (tHSCs) positively participate in human hepatocellular carcinoma (HCC) tumorigenesis and progression. Our previous studies have shown that tHSCs co-culture with dendritic cells (DCs) induced DIgR2 (dendritic cell-derived immunoglobulin receptor 2) expression. The latter is a member of IgSF inhibitory receptor suppressing DCs-initiated antigen-specific T-cell responses. In the current study, we show that hepatic artery injection of DlgR2 siRNA significantly inhibited in-situ HCC xenograft growth in rat livers. Further, 5-FU-medied inhibition of in-situ HCC growth was dramatically sensitized with DlgR2 silence. DlgR2 siRNA injection indeed downregulated DlgR2 in ex-vivo cultured tumor-derived DCs (tDCs). More importantly, tDCs activity was boosted following DlgR2 siRNA. These cells presented with upregulated CD80, CD86 and MHC-II. Production of interleukin-12 and tumor necrosis factor-α was also increased in the DlgR2-silenced tDCs. We propose that DlgR2 knockdown likely boosts the activity of tumor-associated DCs, and inhibits growth of in-situ HCC xenografts.

## INTRODUCTION

Hepatocellular carcinoma (HCC) is a common and lethal malignancy in the world [1–3]. It has been predicted that HCC's mortality rate could be doubled in next decades [4–6]. Tumor immunity has received considerable attentions in the basic research and clinical treatment of HCC [7–9]. Dendritic cells (DCs) are antigen-presenting cells (APC), which are vital in both initiation and regulation of immune responses [10, 11]. Recent studies have focused extensively on the potential function of DCs in tumor immunity [10, 11]. DCs activation is extremely important for proper anti-tumor response [10, 11]. DCs depletion or inhibition, on the other hand, will result in a pro-cancerous environment [10, 11].

It is known that DCs activation is tightly controlled by many inhibitory and stimulatory signal molecule [10, 11]. One key inhibitory protein is DIgR2, or dendritic cell-derived immunoglobulin receptor 2. It is a member of IgSF inhibitory receptor suppressing DC-initiated antigen-specific T-cell responses [12]. Shi *et al*., have previously shown that DCs-specific DIgR2 binds to T cells, causing T-cell hypo-responses [12].

In the process fibrogenesis, tumor-specific hepatic stellate cells (tHSCs) are responsible for the production of extracellular matrix proteins [13–15]. Therefore, the HSCs actively participate in HCC's tumorigenesis and progression [16, 17]. tHSCs could be detected in HCC stroma and peri-HCC tissues, as well as in tumor sinusoids, and the tumor capsule [16–18]. tHSCs participate in a number of key cancerous behaviors, including facilitating extracellular matrix turnover, enhancing growth factor/cytokine signalling, as well as promoting tumor angiogenesis [16–18]. Recent studies have also proposed a novel mechanism of tHSCs in regulating tumor immunity [16–18]. Our recent studies have suggested that tHSCs may directly induce DIgR2 expression in DCs to inhibit T cells (Xia et al., 2017). The current study evaluated its potential effect on HCC growth *in vivo*.

## RESULTS

### DIgR2 upregulation in DCs after tHSCs co-culture

**Figure 1:**
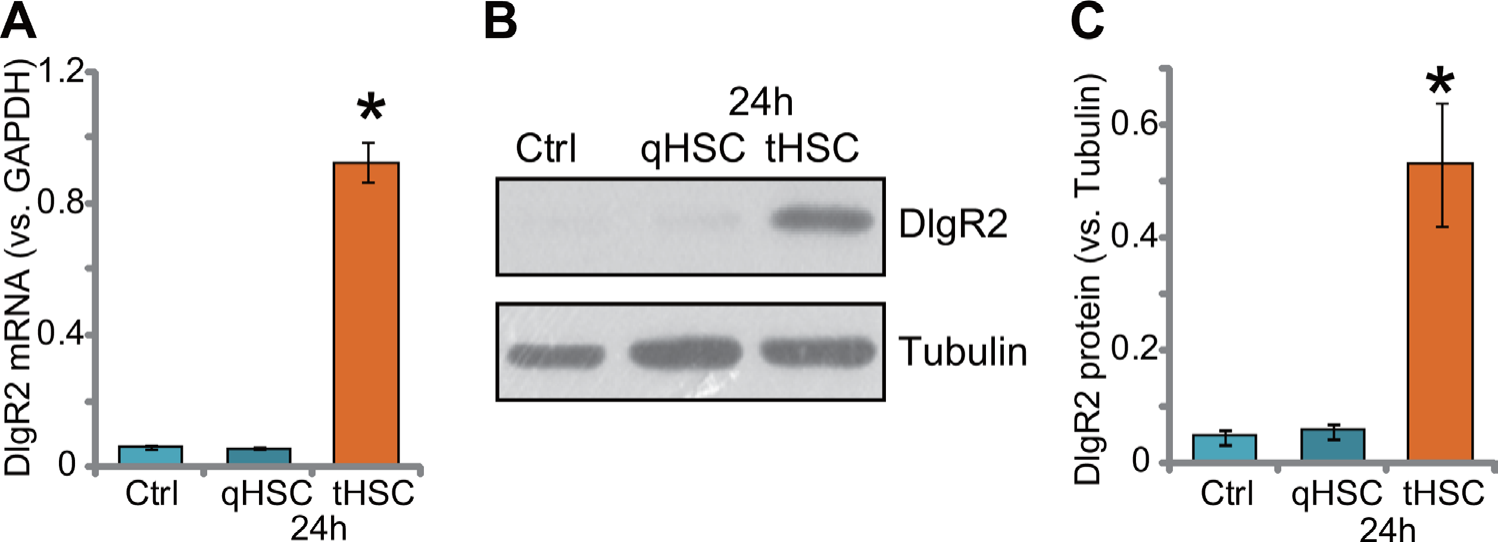
DIgR2 upregulation in DCs after tHSCs co-culture Expression of *DIgR2* *mRNA* (**A**) and protein (**B)**, quantified in (**C**) in bone marrow-derived dendritic cells (mDCs), with/out co-culture of quiescent HSCs (qHSCs) or tumor HSCs (tHSCs), were shown. “Ctrl” stands for mDCs only. “Tubulin” stands for loading control β-Tubulin (Same for all Figures). Data were expressed as mean ± SD (*n* = 5). *P < 0.05 vs. “Ctrl” group. Experiments in this figure were repeated three times, and similar results were obtained.

First, HSCs were derived from control rat livers or in-situ xenograft HCC tissues (See method). They were named as quiescent HSCs (qHSCs) and tumor-specific HSCs (tHSCs), respectively [19]. We wanted to know if priming DCs with tHSCs could induce DlgR2 upregulation (Xia et al., 2017). The bone marrow-derived dendritic cells (mDCs) were derived from the rat femurs using the described method [12]. mDCs were then co-cultured with qHSCs or tHSCs (mDCs to HSCs ratio, 20: 1). After 24 hours, quantitative real-time PCR (“qRT-PCR”) assay results showed that *DlgR2 mRNA* expression level was significantly elevated in tHSCs-primed mDCs, but not in qHSCs-primed cells (Figure 1A). *DlgR2 mRNA* level in mDCs increased over 10 folds with tHSCs co-culture (Figure 1A). Consequently, DlgR2 protein expression in mDCs was also dramatically increased following co-culture with tHSCs (but not qHSCs) (Figure 1B). Quantified blot results in Figure 1C showed about 7–8 fold increase of DlgR2 protein expression in tHSCs-primed mDCs.

### SiRNA-induced knockdown of DlgR2 in tHSCs-primed mDCs

**Figure 2:**
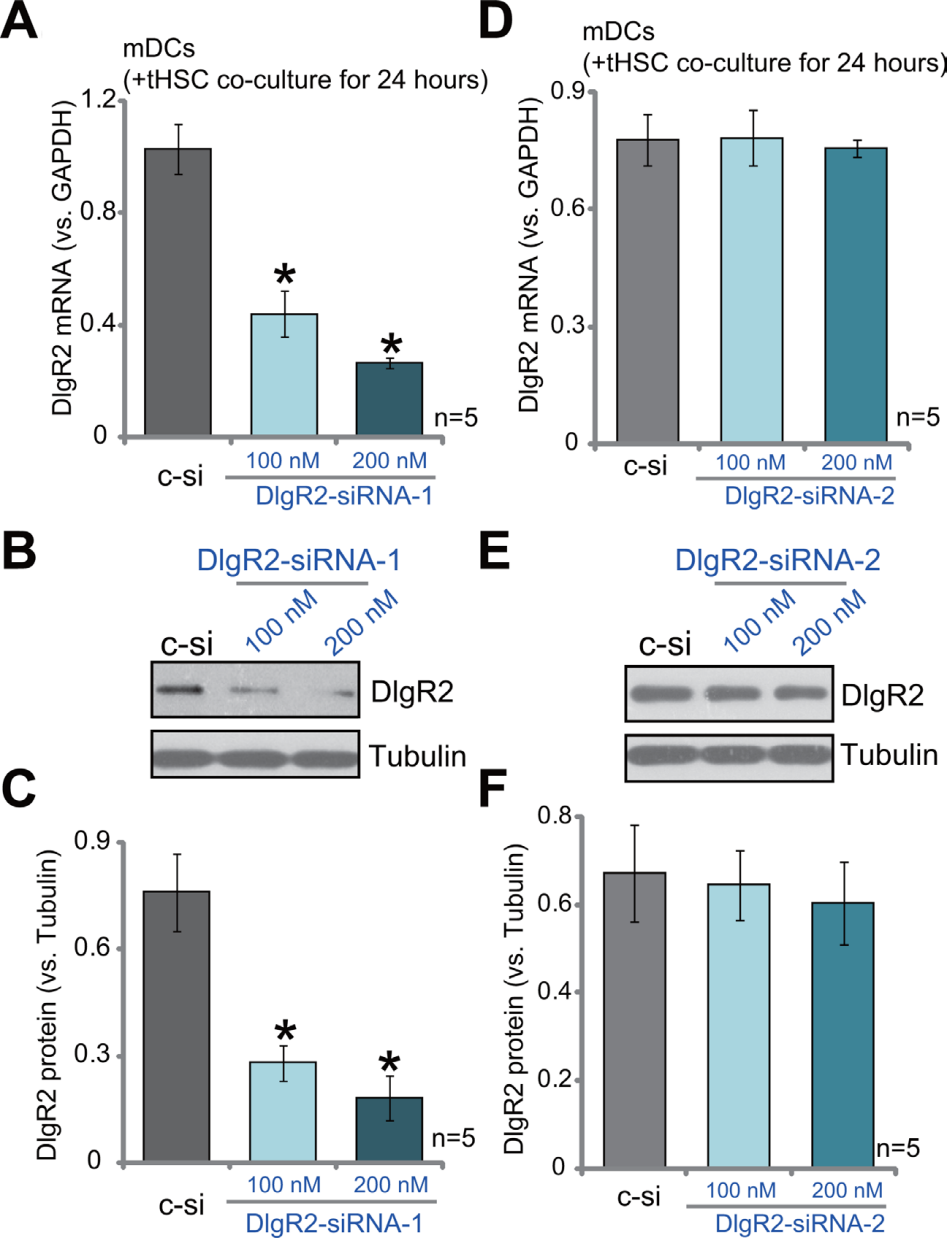
SiRNA-induced knockdown of DlgR2 in tHSCs-primed mDCs mDCs were first co-cultured with tumor specific HSCs (tHSCs) for 24 hours, following by transfection of DlgR2-siRNA-1/2 (at applied concentration) for additional 36 hours; *DIgR2* *mRNA* (**A** and **D**) and protein (**B, C, E** and **F**) expressions were tested. “sc-c” stands for scramble non-sense siRNA control (200 nM). Data were expressed as mean ± SD (*n* = 5). *P < 0.05 vs. “sc-c” group. Experiments in this figure were repeated three times, and similar results were obtained.

The current study aims to understand how DlgR2 expression in DCs could possibly inhibit immunity against HCC. For this purpose, two non-overlapping DlgR2 siRNAs were designed and were transfected to tHSCs-primed mDCs. The two were named as “DlgR2-siRNA-1” and “DlgR2-siRNA-2”. Transfection of DlgR2-siRNA-1, with the sequence of 5′-GAUGGCGUCGGUGAUGGGUTT-3′, efficiently decreased *DlgR2 mRNA* expression in tHSCs-primed mDCs (Figure 2A). Meanwhile, DlgR2 protein expression was also downregulated by DlgR2-siRNA-1 (Figure 2B and 2C). DlgR2-siRNA-1 demonstrated a dose-dependent effect. DlgR2-siRNA-1 at 200 nM was more potent than 100 nM in silencing DlgR2 (Figure 2A–2C). On the other hand, DlgR2-siRNA-2 failed to significantly inhibit expression of *DlgR2 mRNA* (Figure 2D) and protein (Figure 2E and 2F) in tHSCs-primed mDCs. Thus, DlgR2-siRNA-1 was selected for further experiments. Notably, scramble non-sense siRNA control (“sc-c”, 200 nM) didn't change DlgR2 expression in mDCs (Figure 2A–2F).

### DIgR2 siRNA inhibits HCC tumor in-situ growth, and chemo-sensitizes 5-FU

**Figure 3:**
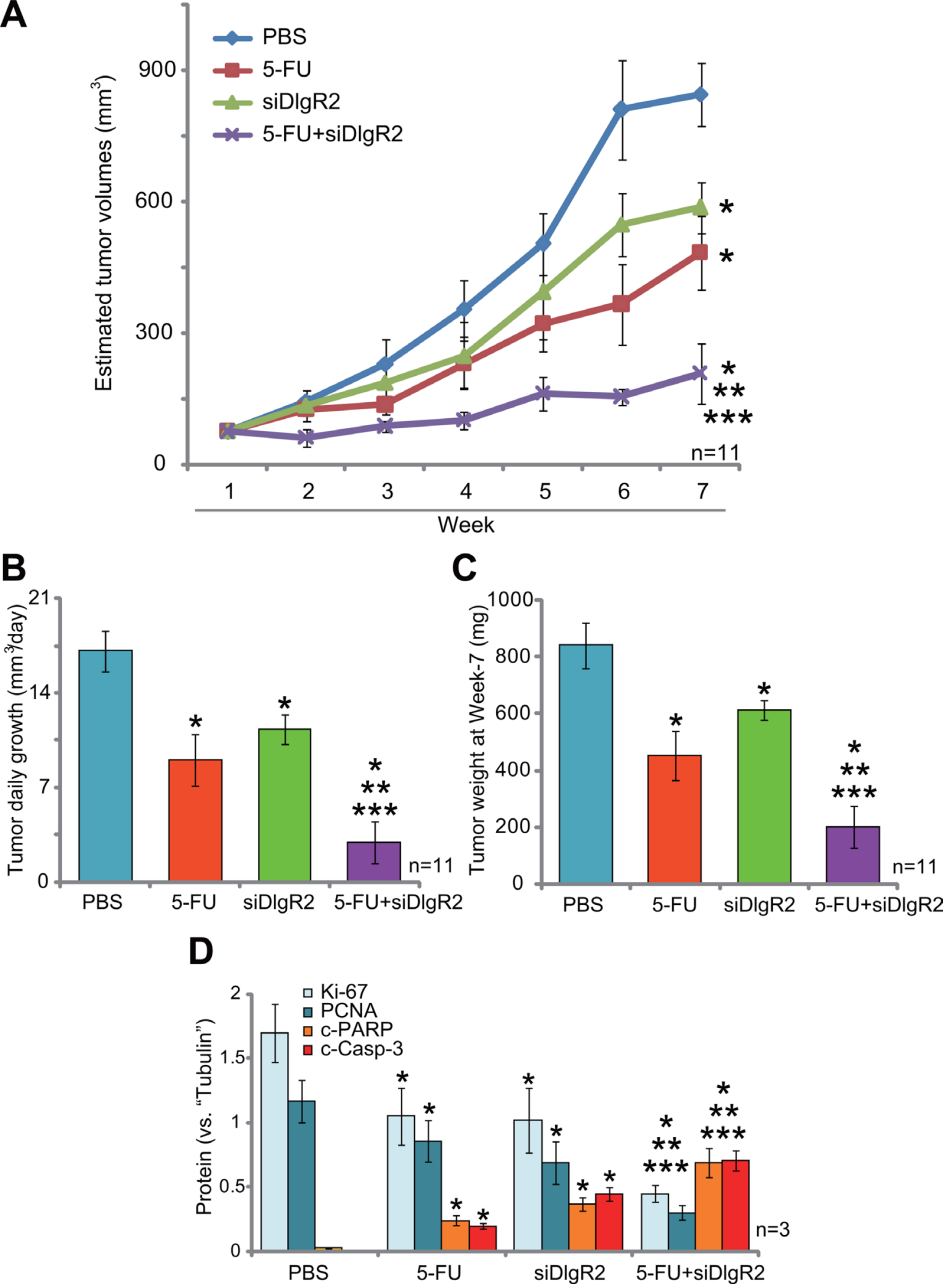
DIgR2 siRNA inhibits HCC tumor in-situ growth, and chemo-sensitizes 5-FU HCC in-situ tumor-bearing rats were subjected to weekly hepatic artery injection of DlgR2-siRNA-1 (“siDlgR2”, 200 pmol, x2/week) and/or 5-FU (5 mg/kg body weight, x2/week), as well as PBS control; Tumor volumes were recorded every week for a total of seven weeks (**A**); Estimated daily tumor growth (in mm^3^ per day) was presented (**B**); At the end of experiment (Week-7), tumors were isolated via surgery and weighted (**C**); Tumor tissues were subjected to Western blotting assay of listed proteins, and blot data of three sets were quantified (**D**). Data were expressed as mean ± SD. *P < 0.05 vs. “PBS” control group. **P < 0.05 vs. “DlgR2-siRNA-1” only group. ***P < 0.05 vs. “5-FU” only group.

DIgR2 is an important inhibitory receptor that shall suppress DCs-induced antigen-specific T-cell responses [12]. Reversely, DIgR2 silence should increase DC functions to inhibit tumor cells. HCC in-situ model was established. As previously described [19], surgery-isolated subcutaneous MRH-7777 HCC tumors were cut into small piece (2 × 1 × 1 mm^3^), and were transplanted to the rat livers to establish the HCC in-situ xenograft model [19]. When the in-situ tumors reached the volumes around 100 mm^3^, rats were subjected to hepatic artery injection of DlgR2-siRNA-1 for two consecutive days. The DlgR2 siRNA injection were repeated every week for a total of 6 weeks. As shown in Figure 3A, DlgR2-siRNA-1 injection inhibited the in-situ growth of HCC xenografts. The estimated tumor volume was lower in the DlgR2-siRNA-1 treatment group, as compared to the control (“PBS” injection) group (Figure 3A). As expected, hepatic artery injection of the chemo-drug 5-FU (5 mg/kg, x2/week, for 6 weeks) also suppressed HCC xenograft in-situ growth (Figure 3A). Remarkably, DlgR2-siRNA-1 significantly chemo-sensitized 5-FU (Figure 3A). Co-administration of DlgR2-siRNA-1 and 5-FU led to profound inhibition of HCC tumor in-situ growth (Figure 3A). The combination was more potent than either single treatment (Figure 3A).

Further analysis showed that estimated daily tumor growth (in mm^3^ per day) was lowest in the combination treatment group (Figure 3B), although each single treatment also decreased daily tumor growth (Figure 3B). When analyzing tumor weight (at the end of experiments, or Week-7), it was again lightest in the combination treatment group (Figure 3C). Notably, rat body weights were not significantly different between the groups. We also failed to detect any signs of apparent toxicities in tested animals. When analyzing tumor tissue lysates (at Week-7), we showed that DIgR2 siRNA and 5-FU synergistically downregulated growth marker proteins, Ki-67 and proliferating cell nuclear antigen (PCNA) (Figure 3D, three sets of blot data were quantified). On the other hand, apoptosis markers, including cleaved-caspase-3 and cleaved-PARP (poly ADP ribose polymerase), were upregulated (see quantified results in Figure 3D). Collectively, these results suggest that DIgR2 siRNA inhibits HCC xenograft in-situ growth, and also chemo-sensitizes 5-FU.

### DlgR2-siRNA-1 injection indeed silences DIgR2 in tumor-derived DCs

**Figure 4:**
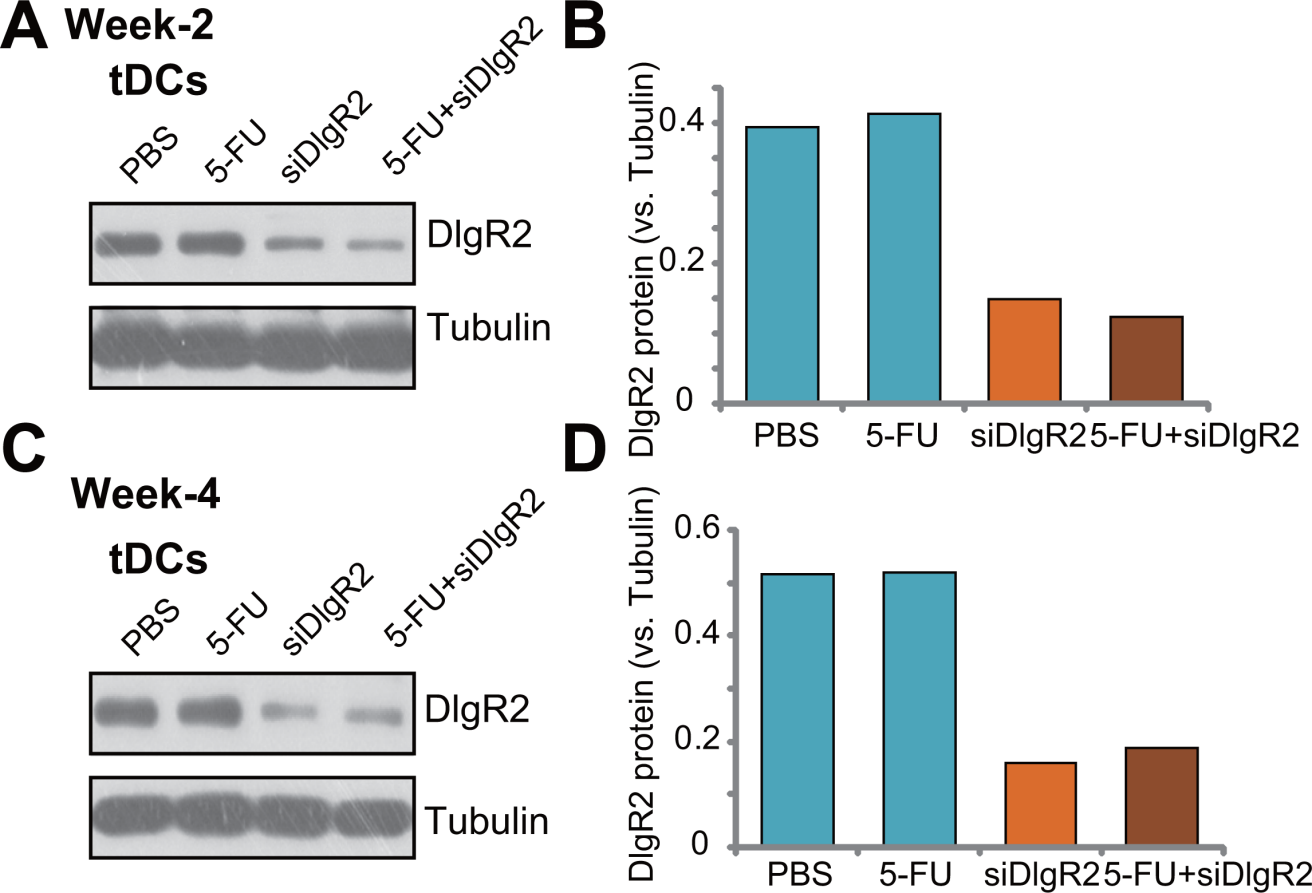
DlgR2-siRNA-1 injection silences DIgR2 in ex-vivo cultured tumor-derived DCs HCC in-situ tumor-bearing rats were subjected to hepatic artery injection of DlgR2-siRNA-1 (“siDlgR2”,, x2/week) and/or 5-FU (5 mg/kg body weight, x2/week), as well as PBS control; At week-2 (day-9) and Week-4 (Day-30), one tumor per group was isolated; Tumor-specific DCs (tDCs) were isolated and primary cultured; Expression of listed proteins were tested (**A** and **C**), and data were quantified (**B** and **D**). Experiments in this figure were repeated three times, and similar results were obtained.

In order to confirm that DIgR2 was indeed silenced by the DlgR2-siRNA-1 injection *in vivo*. Dendritic cells were isolated from the HCC xenograft tissues and cultured *ex-vivo* (see METHODS), which were named as tDCs. As shown in Figure 4A, expression of DIgR2 was relatively high in the *ex-vivo* cultured tDCs from control HCC tumors (at Week-2). Significantly, its expression level was downregulated in tDCs-derived from DlgR2-siRNA-1-treated HCC tumors (Figure 4A). Quantified results in Figure 4B showed that DlgR2-siRNA-1 injection caused over 60% of downregulation of DIgR2 protein in *ex-vivo* cultured tDCs. Similar results were also obtained from tDCs that were derived from tumor tissues at Week-4, and DlgR2-siRNA-1 injection significantly downregulated DIgR2 in tDCs (Figure 4C and 4D). It should be noted that 5-FU treatment didn't change DIgR2 protein expression in the tDCs (Figure 4A–4D). These results confirm that injection of DlgR2-siRNA-1 silenced DIgR2 in tDCs.

### DlgR2 siRNA boosts function of tumor-derived DCs

**Figure 5:**
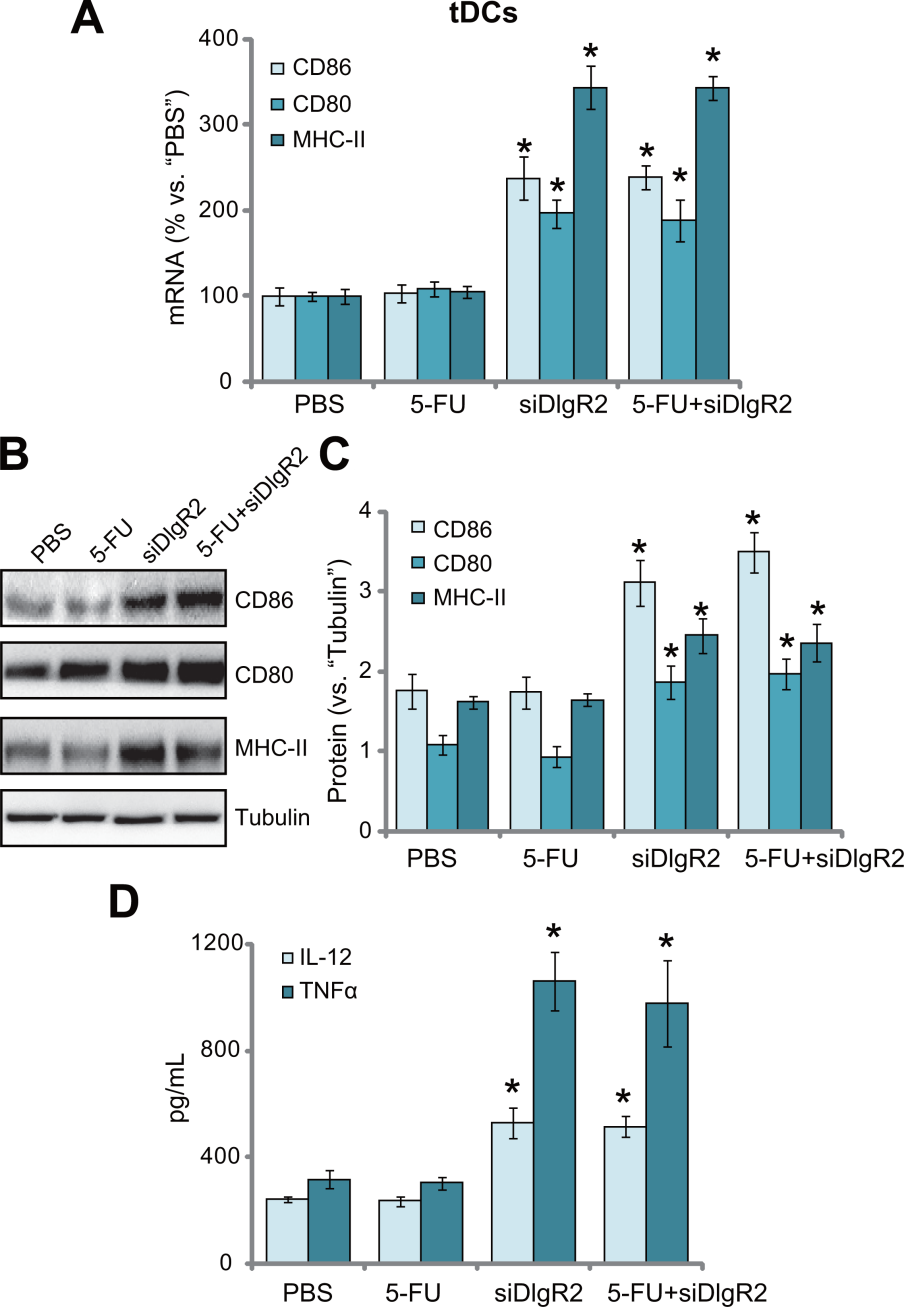
DlgR2 siRNA boosts function of tumor-derived DCs The *ex-vivo* cultured tumor specific DCs (tDCs) were subjected to qRT-PCR assay (**A**), Western blotting assay (**B**), and quantified in (**C**) and ELISA assay (**D**) of listed proteins (A-C) and cytokines (D). Data were expressed as mean ± SD. *P < 0.05 vs. “PBS” control group. Experiments in this figure were repeated three times, and similar results were obtained.

We next tested the potential function of the tumor-derived DCs (tDCs). The B7-related cell surface proteins or co-stimulatory molecule CD80 (B7–1) and CD86 (B7–2) are expressed on DCs, which respectively binds to the homologous T cell receptors CTLA-4 and CD28, leading to T cell activation [20, 21]. Major histocompatibility complex (MHC) class II (MHC-II) expression in DCs is also critical in antigen presentation and cellular immune responses [22–24]. The qRT-PCR assay results in Figure 5A showed that, as compared to the control tDCs, mRNA expressions of the co-stimulatory molecule (CD86, CD80 and MHC-II) were significantly increased in *ex-vivo* cultured DlgR2-silenced tDCs. Further, CD86, CD80 and MHC-II protein expressions were also increased in DlgR2-silenced cells (Figure 5B and 5C). DC-associated cytokines were also tested. ELISA assay results confirmed that tumor necrosis factor-α (TNF-α) and interleukin-12 (IL-12) productions were also boosted following DlgR2 knockdown in *ex-vivo* cultured tDCs (Figure 5D). Notably, treatment with 5-FU failed to inhibit the functions of the tDCs (Figure 5A–5D). Together, these results suggest that DlgR2 siRNA boosts function of *ex-vivo* cultured tDCs.

## DISCUSSION

DCs function could be controlled by a number of inhibitory and excitatory factors [10, 11, 25, 26]. DIgR2 is novel and important member of IgSF inhibitory receptor [27–29]. DigR2 inhibits DC-initiated antigen-specific T-cell responses [12, 27–29]. DIgR2 has at least two immuno-receptor tyrosine-based inhibitory motifs (ITIMs) in the cytoplasmic region [12]. It associates with tyrosine phosphatase protein SHP-1, the latter is a Src homology-2 (SH2) domain-containing protein. DC-derived DIgR2 binds to yet unknown receptor in T cells, causing T-cell proliferation inhibition and hypo-responses [12]. On the other hand, DIgR2 inhibition, by pretreatment with DIgR2-Ig fusion protein or targeted-siRNA, boosted DC-provoked T-cell proliferation and antigen-specific T-cell responses [12].

In the current study, we found that DIgR2 expression was significantly elevated in DCs-derived from in-situ HCC xenografted (tDCs). Significantly, hepatic artery injection of DlgR2 siRNA inhibited in-situ HCC xenograft tumor growth in rats. Further, 5-FU-medied suppression of HCC tumor in-situ growth was also dramatically sensitized by DlgR2 silence. Remarkably, hepatic artery injection of DlgR2 siRNA indeed downregulated DlgR2 in *ex-vivo* cultured tDCs. More importantly, tDCs activity was boosted following DlgR2 silence, presenting with upregulation of CD80, CD86 and MHC-II, as well as over-production of IL-12 and TNF-α. We thus propose that silence of DlgR2 boosts the activity of tumor-associated DCs, thus likely inhibiting HCC tumor in-situ growth.

For the mechanism of DlgR2 upregulation in tDCs, we proposed that tHSCs could be at least one cause. Our studies [19, 30] and others have implied an important function of tHSCs in inhibiting tumor immunology [16–18]. For example, we found that tHSCs could inhibit T cell proliferation and induce T cell apoptosis [19]. Further, tHSCs also induce T cell hypo-response [30]. In line with our previous findings, we showed that tHSCs co-culture induced significant upregulation of DIgR2 (both mRNA and protein) in mDCs. Thus, we propose that tumor-specific/activated tHSCs induce DIgR2 expression to inhibit DCs, therefore likely causing immuno-depression against HCC cells. On the other hand, DIgR2 silence re-stores DCs function and inhibits HCC tumor growth.

Immune evasion of HCC and other tumors [7–9] is considered as a characteristic hallmark of cancer [9, 31]. Studies have confirmed that both the number and activity of anti-tumor immune cells, including DCs and tumor-killing T cells, are decreased at the tumor site and in the lymphoid organs [9, 31]. Our results propose that tHSCs (or other unknown mechanisms)-induced upregulation of DIgR2 in DCs could be the cause of immune suppression in HCC. DIgR2 silence, on the other hand, booted tDCs function and inhibited HCC xenograft in-situ growth.

## MATERIALS AND METHODS

### Chemicals, reagents and antibodies

The antibodies of this study were obtained from Abcam (Suzhou, China) and Cellular Signaling Tech (Nanjing, China). The reagents for cell culture were purchased from Invitrogen (Shanghai, China). 5-FU was obtained from Sigma (Nanjing, China). The mRNA primers were synthesized by Genepharm Company (Shanghai, China).

### Rat HCC tumor *in-situ* model

The buffalo rats (4–5 week-old) were maintained at the Animal Center of Anhui Medical University (Hefei, China). The detailed protocols of in-situ HCC xenograft tumor rat model was described previously [19, 30]. Briefly, the MRH rat HCC cells [19, 30] were initially injected *s.c*. to the flanks of the rats. Four weeks after the initial injection, xenograft tumors (around 100–150 mm^3^ in volume) were established and were surgery isolated. The fresh HCC xenografted were then cut into small pieces (2 × 1 × 1 mm^3^), and were transplanted to the livers of the rats [19]. Tumor extension was allowed for another 2–3 weeks and were detected by ultrasound. Afterward, rats were randomized into four groups, and were subjected to weekly hepatic artery injection of DlgR2-siRNA (200 pmol, x2/week) and/or 5-FU (5 mg/kg body weight, x2/week), or the PBS control; Injection was performed at the first two days of the week. The size of the tumors was measured by caliper every week, and estimated tumor volume was calculated using the following formula: π/6×width ^2^× length. Estimated daily tumor growth was calculated by: (estimated tumor volume at Day-42 deducting tumor volume at Day-1)/42 [32, 33]. The animal protocols were approved by Institutional Animal Care and Use Committee (IACUC) and Ethics Board of Anhui Medical University. All surgical procedures were performed with anesthesia. All efforts were made to minimize suffering.

### Culture of tumor-associated DCs (tDCs) from in-site HCC tumor tissues

The fresh in-situ HCC xenograft tissues were perfused at a flow rate of 10 mL/min with Gey's balanced salt solution (GBSS) for 10 min, followed by 100 mL of 0.12% pronase E (Roche) dissolved in GBSS for another 10 min [19, 30]. The HCC tissues were then excised, dissected and incubated for 30 min with continuous shaking, with 0.04% pronase E, 0.05% collagenase and 0.002% DNase I (Sigma) in 100 mL GBSS. After digestion, the cell suspension was passed through a 0.22-μm mesh and centrifuged at 500 × g for 10 min. Subsequently, cells were purified with 8% Nycodenz (Sigma) gradient centrifugation. The resulting monocytes were then sorted by flow cytometry with anti-CD11c antibody (Abcam, Suzhou, China). Purified DCs were then generated via culturing in 6-well tissue-culture plates (Costar) plus 50 ng/mL GM-CSF and 10 ng/mL IL-4 (1,000 U/mL, R&D systems) for 5 days in RPMI 1640 FCS medium (no antibiotic). On day-5, DCs were determined by flow cytometry with anti-CD11c antibody (over 90% positive rate).

### Isolation and culture of HSCs

As described previously [19, 30], HSCs were derived from the normal liver tissues or in-situ HCC tissues of Buffalo rats. The livers were subjected to perfusion and digestion via the described method [19, 30]. Thereafter, the resulting cell suspensions were purified by centrifugation through a 8% Nycodenz (Axis-Shield PoC) gradient. The achieved HSCs were cultured in DMEM plus FBS medium. Trypan blue exclusion was applied to test cell viability (always over 90%). The desmin immuno-staining assay was performed to determine the purity of quiescent HSCs (qHSCs) and tHSCs, ranging over 85% [34].

### Primary culture of bone marrow dendritic cells (mDCs)

As described [19, 35], bone marrow cells from the Buffalo rat femurs were flushed and cultured in the RPMI-1640 medium, plus rat GM-CSF (50 ng/mL, Sigma) and IL-4 (1,000 U/mL, R&D systems). The non-adherent cells were released spontaneously from the proliferating cell clusters, harvested, washed, and resuspended in the medium described previously [35].

### mDCs and HSC co-culture

For each well, 2.5 × 10^5^ mDCs were co-cultured with 1.25 × 10^4^ HSCs (20: 1, mDCs to HSCs) for applied time.

### DIgR2 siRNA

Two distinct DIgR2 siRNA (named as “DIgR2-siRNA-1/-2”) were designed and provided by the Genepharm Company. The siRNA (100/200 nM) was transfected to mDCs by the Lipofectamine 2000 reagent (Invitrogen, Shanghai, China). DIgR2 downregulation was confirmed by qRT-PCR assay and Western blotting assay.

### RNA extraction and real-time PCR

Cellular RNA was extracted via TRizol reagents (Promega) [36]. The reverse transcription was performed using the SYBR Green kit (Applied Biosystems) [37–39]. The ABI-7600 Fast Real-Time PCR system was utilized to perform the quantitative real time-PCR (qRT-PCR) assay [40, 41]. For each analysis, melt curve analysis was performed to calculate the melting temperature of the product. *GAPDH* (*glyceraldehyde-3-phosphatedehydrogenase)* mRNA was always tested as the reference gene. The 2^−ΔΔCt^ method was applied to quantify targeted mRNA change [40, 41]. *DIgR2 mRNA* and *GAPDH* *mRNA* primers were described previously [12, 40, 41].

### Western blotting assay

For each condition, lysate samples (30 μg per lane) were separated by the 10% SDS-PAGE gels, and were transferred to the PVDF membranes [39] (Millipore, Suzhou, China). The blots were blocked and incubated with designated primary/secondary antibodies. Enhanced chemiluminescence (ECL) detection reagents were then utilized to visualize the band/s via x-ray film exposure. The intensity or the total gray of each band was quantified by the ImageJ software.

### Enzyme-linked immunosorbent assay (ELISA) assay

Cytokine production in the conditional medium was tested via the corresponding ELISA kit (R&D Systems), using the attached protocol [42].

### Statistical analysis

All statistical analyses were conducted using SPSS 15.0 software. Data were always expressed as the mean ± SD (standard deviation). *P* < 0.05 was considered as statistically significant different.

## CONCLUSIONS

DlgR2 knockdown likely boosts the activity of tumor-associated DCs, and inhibits growth of in-situ HCC xenografts. DC-derived DlgR2 could be a novel oncotarget for HCC treatment.
